# Propofol: A Medication That Changed Pediatric Anesthesia Practice

**DOI:** 10.1111/pan.70001

**Published:** 2025-06-21

**Authors:** Brian J. Anderson, L. Ignacio Cortinez

**Affiliations:** ^1^ Department of Anaesthesiology University of Auckland Auckland New Zealand; ^2^ División Anestesiología, Escuela de Medicina Pontificia Universidad Católica de Chile Santiago de Chile Chile

**Keywords:** anesthesia, intravenous, pediatrics, pharmacodynamics, pharmacokinetics, propofol, target‐controlled infusion, TIVA

## Abstract

The introduction of propofol changed our understanding of pediatric anesthesia pharmacology, research approaches for intravenous drug study, and led to infusion pump development for the maintenance phase of anesthesia. The story of propofol development provides a blueprint for other intravenous drug development. The introduction of the target concentration strategy based on pharmacokinetic‐pharmacodynamic and covariate understanding is central to total intravenous anesthesia techniques and is best exemplified by propofol. While use of the EEG to gauge depth of anesthesia preceded the propofol era, processed EEG signals enabled description of the relationship between propofol plasma concentration and effect, advancing propofol use and safety. Clinical need drove the characterization of propofol pharmacokinetics and concentration effect relationships in children. Subsequently, study in populations such as neonates, the critically ill, and children with obesity explored covariate influences. Target‐controlled infusions also required an appreciation of effect site concentrations and time delays, and drug interactions such as those between propofol and opioids. Supraglottic airway use in children paralleled propofol use because greater depression of pharyngeal and laryngeal reactivity was noted with propofol than seen than with thiopental. Environmental concerns with the carbon footprint of inhalational agents may yet see infusions assume dominance for maintenance anesthesia.

## 1 Introduction

The introduction of propofol into clinical anesthesia had a major influence on practice. The drug rapidly assumed dominance over other intravenous options for the induction of anesthesia. Propofol use for maintenance does not yet rival that of the inhalational agent, sevoflurane, but acceptance is increasing [1], even in patients with complex physiology such as premature neonates or those nursed in intensive care units. None of the other hypnotics currently available have successfully competed with propofol in adults or children [2].

This ascendency of propofol was associated with new concepts to steer clinical pharmacology interpretation. Whether serendipity or design, both the ability to analyze complex pharmacologic data and cerebral effect monitoring underwent advances that allowed improved methods to interpret pharmacokinetics and pharmacodynamics (PKPD) of anesthesia drugs. The development of programmed infusion pumps capable of incorporating PKPD knowledge improved the ability, efficacy, and safety of drug delivery. Propofol was the drug spearheading these innovations in anesthesia research and practice.

## 2 Propofol Development

Propofol development was led by John Glen, a veterinary medicine graduate from Glasgow University, Scotland [3]. Investigation initially centered on lipophilic compounds that could be prepared in aqueous solution with the aid of cremophor (e.g., propanidid; Bayer, Ludwigshafen, Germany), a mixture of polyoxyethylated triglycerides that acts as a nonionic tensioactive agent for poorly water‐soluble pharmacological agents. However, the realization that this carrier caused anaphylaxis led to the use of an emulsion formulation containing 10% soybean oil and 1.2% purified egg phosphatide. Subsequent collaboration between physicians and pharma proved beneficial for development. An early population‐type pharmacokinetic analysis of this emulsion formulation of propofol was undertaken using a nonlinear least‐squares regression analysis. Parameters estimates (and standard deviations) for a three‐compartment open mammillary model were described with exponential equations [4]. That study included infusion data because it had become clear that maintenance anesthesia was possible with propofol; the drug was more than just an induction agent. The infusion pumps available at the time were incapable of delivery at rates required to maintain anesthesia. Better pumps were required [5]. Approvals to market the drug for induction and maintenance anesthesia for up to 1 h were obtained in 1986. The Food and Drug Administration, USA, approved the drug later in 1989 although it still remains off‐label for children younger than 3 years of age. Clinical investigation in 1988 established infusion rates for maintenance of anesthesia in adults whereby the rate was gradually reduced to compensate for redistribution of the drug over time (10, 8, and 6 mg/kg/h scheme) [6]. Similar regimens for children [7] were not possible before a better understanding of pediatric pharmacology was reached. That understanding came from the use of population modeling. Table 1 displays a timeline of events associated with propofol ascendency.

**TABLE 1:** A timeline of events associated with propofol ascendency.

Year	General event	Propofol related events
1936	EEG used to monitor drug effects [8]	
1977		Propanidid (cremophor EL)
1979	PKPD approach for NMBDs [9]	
1981	PKPD models for dose response effects [10]	
1983	The laryngeal mask [11]	
1984	Population modeling [12]	
1986		Propofol marketing approval UK and Europe
1987		Propofol pharmacokinetics described [4]
1988		Adult manual propofol infusion regimen [6] Propofol effects on airway described [13]
1989		FDA approval of propofol in USA
1990	Ohmeda 9000 Syringe pump [5]	
1991	Mixed effects models popularized [14]	
1992	Context sensitive half‐time described [15]	
1994	Bispectral Index Monitor [16]	Population modeling propofol pharmacokinetics in children [17]
1995	Target concentration approach [18]	
1997	Allometry used in pediatric pharmacokinetic exploration [19]	Nomenclature for target‐controlled infusion (TCI) pumps [20]
1998		Diprifusor infusion pump [21]
1999		Manual infusion pediatric regimen [7] Bispectral Index used to describe propofol response [22]
2002	Allometry for size scaling in pediatric anesthesia [23]	Exploration propofol pharmacokinetics in critically ill children [24]
2003		Propofol–remifentanil interactions in adults [25]
2004		Surface pharmacodynamic interaction models used for propofol [26] Exploration of dose in neonates and infants [27]
2007		Propofol clearance maturation in infants [28]
2008	TIVA use in children increases [29]	
2010		Effect fat mass on propofol adult PK [30]
2013	Maturation and size standards for pediatric pharmacokinetics [31]	
2014		Allometry used for scaling in universal propofol pharmacokinetic model [32]
2015		Pharmacokinetics–pharmacodynamics for obese children described [33]
2017	Fat mass as a covariate for modeling [34]	
2016	pEEG standard for monitoring during TIVA (with NMBDs) [35]	
2018		Propofol–remifentanil pharmacodynamic interactions in children [36] Link parameter (*T*_1/2_keo) scaled using allometry [36]
2019		Propofol clearance maturation in children [37]

Abbreviations: NMBDs, neuromuscular blocking drugs; pEEG, processed ECG; PKPD, pharmacokinetics–pharmacodynamics; *T*_1/2_keo, equilibration half‐time between plasma and effect compartment.

## 3 The Rise of Population Modeling

The use of population modeling was hugely influential, enabling both a better understanding of propofol PKPD and better clinical use of the drug. Familiarity with this technique allowed exploration of pharmacology aspects (e.g., target centration approaches, developmental pharmacology, covariate effects, drug–drug interactions, drug hysteresis explanation, and drug–response relationships) that not only benefited our routine use of propofol, but also set up blueprints for exploration of other drugs. However, these pharmacological advances gained from population modeling of propofol are unrecognized by many anesthesia practitioners. Target‐controlled infusion pumps are programmed with models that have benefited from population modeling, but the models themselves remain a mystery to many users of those pumps.

The propofol era coincided with the ascendency of population modeling to describe PKPD relationships and associated variability [38]. Anesthesiology investigators, both adult and pediatric, were enthusiastic proponents of this type of modeling for the investigation of propofol, its covariates, interactions with other drugs, effect on sedation, cardiovascular consequences, respiratory physiology, disease progression, pain resolution, and placebo effects [39]. These pioneers improved not only PKPD understanding of the drug but also improved the use of population modeling to investigate aspects of pharmacology far beyond simple pharmacokinetics. Population modeling in anesthesia moved from exponential equations to compartment models described using the parameters, clearance (CL) and volume (*V*), with differential equations; a tool that could be performed on desktop computers with powerful computational ability. This digital age coincided with propofol development.

### 3.1 Mixed Effects Models

Much of the research into propofol PKPD has been undertaken using nonlinear mixed effects models (e.g., NONMEM, ICON Development Solutions, Maryland, USA). Mixed effects models account for variability between subjects. The Holy Grail of clinical pharmacology is the prediction of drug PK and PD in the individual patient, and this requires knowledge of the covariates that contribute to variability [40]. If variability between patients is modeled, then the magnitude of the difference between predictions and the observations can be predicted in the next subject. Covariate information (e.g., weight, age, pathology, pharmacogenomics) is used to describe predictable sources (fixed effects) of variability. Random effects explain unpredictable variability (e.g., parameter variability and residual error). Mixed effects models contain both fixed and random effects.

Population models have proven useful for pediatric studies because they are flexible with sparse data, data can be pooled across studies, sampling times are not crucial, and children with missing data can be included in the analysis [41]. PKPD models can be directly translated into clinical anesthesia: target‐controlled infusions (TCI) are programmed with PKPD parameter sets, drug interaction models describe the effects of drugs given in combination, and simulation studies are used to optimize study design and drug dosing.

**FIGURE 1:**
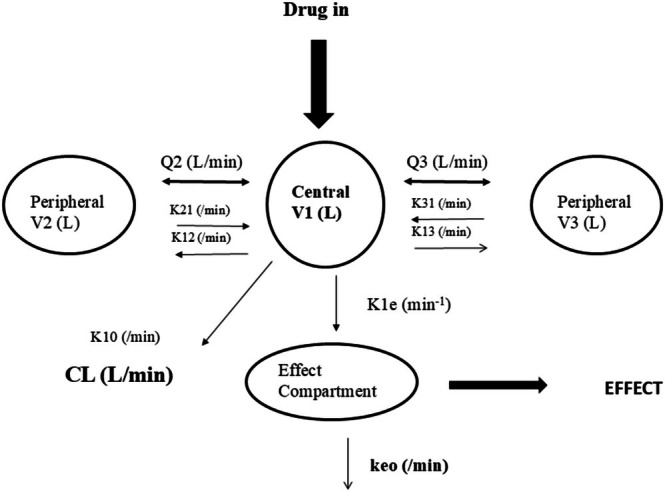
A three‐compartment model with an additional compartment used to describe concentration in the effect compartment. A single first‐order parameter (*k*1e = keo at steady state) describes the equilibration rate between the central and effect compartment. The letter *k* denotes rate constants. Parameters such as between‐compartment clearance (*Q*), elimination clearance (CL), and volume (*V*) are used to describe drug disposition.

Population modeling using compartment (Figure 1) analyses to describe pharmacokinetics in children rapidly followed that in adults. An early model in children compared traditional approaches such as naïve or simple two‐stage methods [42] with a population method [17]. The population model was used for clinical use because there was a clinical need to understand covariates (e.g., age and size) and how they impacted dose. Subsequently, studies in populations such as neonates, the critically ill, and children with obesity broadened clinical use by further exploring covariate influences [24, 33, 43, 44, 45].

Pharmacokinetic modeling skills developed during the characterization of propofol disposition were extended to multiple other drugs used in anesthesia. The need to predict dose in very young children led to investigation of pharmacokinetic and pharmacodynamic changes that occur after birth in that population.

### 3.2 Developmental Pharmacology

**FIGURE 2:**
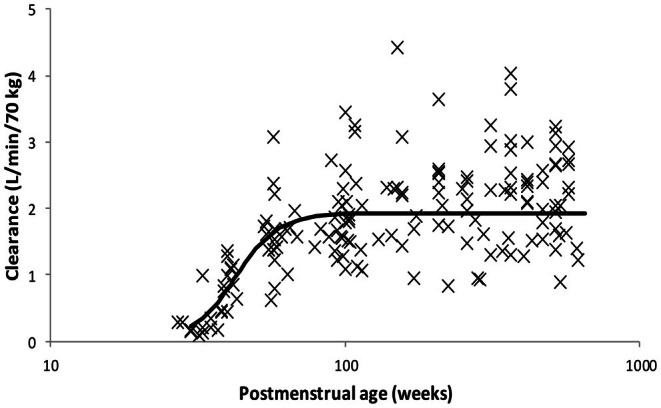
Maturation of propofol clearance with age in children. The population prediction is shown as a solid line. Individual estimates demonstrate variability evident despite correction for the covariates of age and size. From Morse et al. [37], used with permission.

The major differences between children and adults concern growth and size. Most enzyme systems responsible for metabolic clearance are immature at birth and mature within the first few years of life. Propofol has served as a drug template to explore developmental pharmacology. Immaturity of clearance pathways, variability, and subsequent maturation was initially characterized using pooled propofol data [28, 46]. Population modeling using an extension of this pooled dataset has been used to describe clearance maturation for propofol (Figure 2) [37]. Clearance changes were described using a maturation function related to age expressed as postmenstrual rather than gestational or postnatal. Events around birth may also have influence on subsequent clearance maturation [47], and covariates such as gestation and postnatal age have been explored using propofol [48].

Clearance changes after maturation can be attributed to the nonlinear relationship between size and function using allometric theory [23, 49, 50]. Anesthesiology researchers were the first to explore allometric scaling in children [19, 23] and it is now a major consideration for adults as well, pioneered in propofol [32]. Models using allometry with consideration of fat mass (i.e., total body mass minus fat free mass although actual body weight minus lean body weight commonly used) [34] were undertaken using propofol [30]. New pharmacological constructs (e.g., context sensitive half‐time), with propofol as an example, have been introduced to explain behavior in both adults [15] and children [51].

This standardization of size and maturation has proven popular [31] and is useful for comparing different studies, rationalizing dose prediction in patients of all ages, detecting errors, and learning about biology [52]. An appreciation of drug clearance maturation profiles led to questions about the maturation of those enzyme systems responsible for clearance pathways [53], about the role of pharmacogenomics [54] and its contribution to variability [55], and renal function maturation [56].

### 3.3 Reducing Parameter Variability

A key feature of population modeling is that it explores variability. Covariates that contribute to that variability can be identified using population modeling and factored into TCI pumps to reduce variability of parameter estimates (e.g., CL, *V*). The ability to find the dose that can be used in any one individual requires an understanding of covariate influences.

Propofol has served as an example for exploration of covariate influences. The importance of weight is well established, although allomeric scaling of that weight in adults is more recent [57]. The impact of age in adults is recognized [22] but contributors to that age effect remain slightly more elusive; pathology, disease state, clearance, pharmacodynamic sensitivity, and even “frailty” has been evoked as influential. Maturation of propofol clearance systems in the very young is well‐documented and used in TCI pump programs.

The clinical expectation that use of age and size will increase the predictive accuracy of TCI pumps remains untrue because those two covariates only explain part of the parameter variability [58]. Pumps are programmed with median estimates of parameters and patients may not be adequately sedated because those median parameter estimates are not appropriate for an individual. The clinical use of propofol infusion has taught practitioners the impact of parameter variability and the importance of covariates.

It is not yet possible to individualize dose based on covariate influences. Consequently, cerebral monitoring is required to gauge the level of consciousness, and dose can be titrated against this consciousness level. This was thought unnecessary for inhaled anesthesia agents because end‐tidal vapor concentration monitoring is possible. The concentration at which 50% of patients are adequately anesthetized (*C*_50_) of sevoflurane is associated closely with minimal alveolar concentration (MAC) measures [59]. However, pharmacokinetic parameter variability with inhaled agents is also large, and there is also considerable variability in pharmacodynamic parameters [60].

### 3.4 Drug Interactions

**FIGURE 3:**
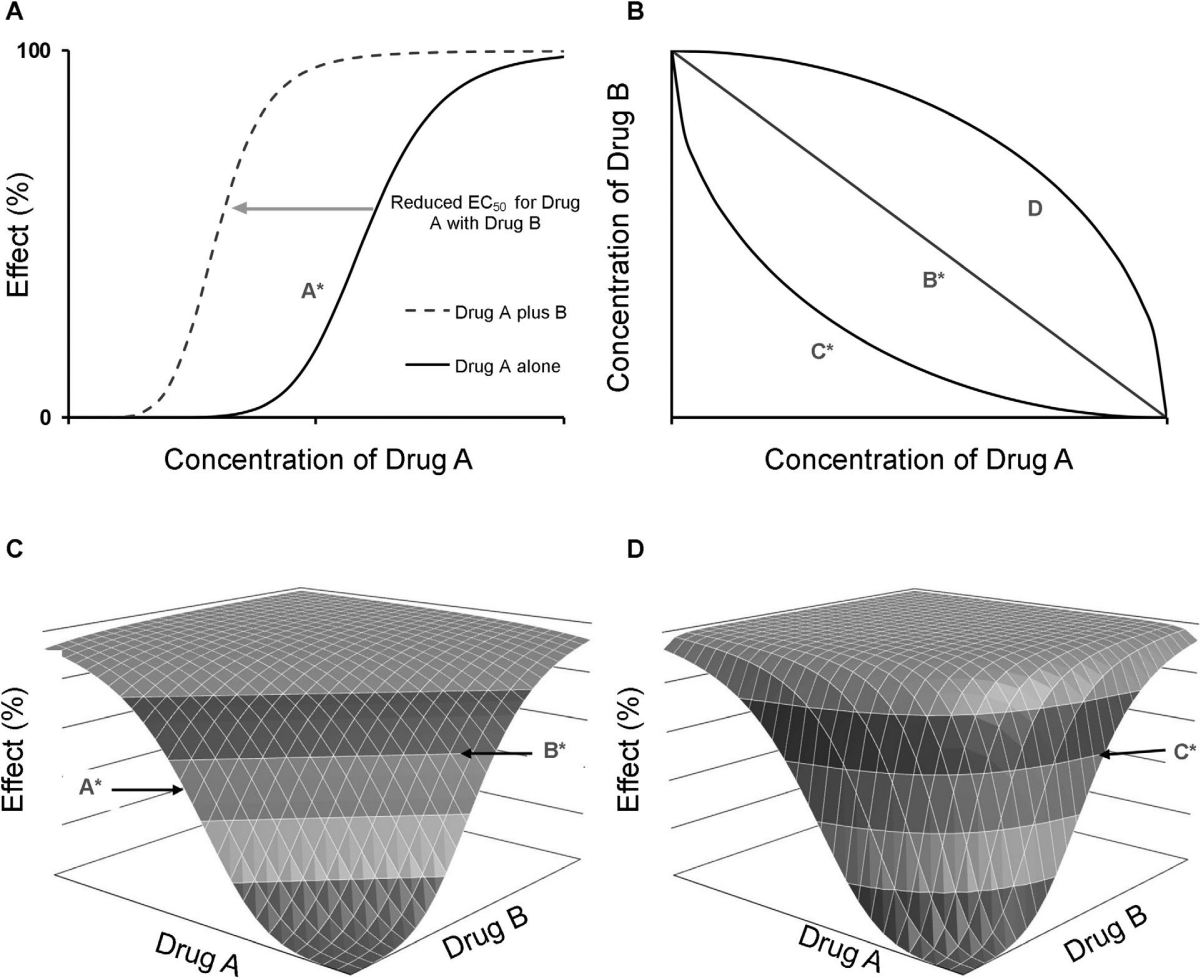
Methods of investigating interactions. (A) Shift in response curve analyses involve plotting the concentration‐ (or dose‐) effect relationship for one drug alone (A*) and in the presence of steady‐state concentrations of a second drug. (B) Isoboles are constructed using iso‐effect lines with curves derived from observations assessed against the expected (or “additive”) response line (B*). Supra‐additivity is depicted by curves bowing toward the plot origin (C*), whereas infra‐additivity is shown with outward curves (D*). Information from both methods is represented within response surfaces with isoboles displayed as horizontal planes and individual concentration–response curves as vertical slices (indicated by arrows on surfaces for A* single concentration–response curve drug A, B* additive isobole, and C* supra‐additive isobole). (C) Additive response surface for two drugs. (D) Synergistic response surface for two drugs, with synergy depicted through outward bowing of the surface. From Hannam and Anderson [64].

Drug interactions may involve PK interactions, PD interactions, or even both [61, 62] and their inclusion in PKPD models has increased applicability and usefulness and provides the opportunity to describe the time course of multiple drug effects [63, 64]. The population approach to PD interaction models often uses a “response surface” (Figure 3); models introduced by anesthesia researchers that commonly used propofol and an opioid [65, 66]. An example is the interaction between propofol and remifentanil in adults [26, 67]; interactions that were subsequently explored in children [36, 68]. These results supported only a modest effect of remifentanil on the bispectral index (BIS) at clinically relevant concentration ranges, encouraging further exploration of electroencephalographic signals that might better monitor analgesic effect. These studies also led to the successful use of combination therapy during the maintenance phase of anesthesia.

## 4 Use for Maintenance Anesthesia

Propofol proved to be a very good drug for both the induction and the maintenance phases of anesthesia. Original approvals in 1988 included maintenance anesthesia for up to 1 h. Propofol is now extensively used for total intravenous anesthesia (TIVA). It is also widely used for intravenous sedation, so much so that it is used as a comparator for new drugs used for sedation [2, 69, 70].

Programmable, portable infusion pumps for delivery of propofol are now common. They were developed from work involving a computer, programmed with a pharmacokinetic model, determined and instructed as an infusion pump to deliver an appropriate infusion rate that rapidly achieved and maintained a desired drug concentration [71]. The need for pumps that could deliver propofol to achieve a specific target concentration led to both the development of these pumps and investigation of pharmacokinetic parameter sets to populate them. Propofol was the prototype drug used in these pumps. Current infusion pumps are populated by parameter sets for multiple drugs.

### 4.1 Parameter Sets

PKPD modeling has had a major role in identifying dose, interactions, and adverse effects of drugs used for TIVA. Propofol is the principal drug used for TIVA, and propofol parameters sets (i.e., models) are programmed into infusion pumps. TIVA also requires an appreciation of drug interactions such as those between propofol and opioids [67, 72]. Algorithms built on PKPD models permit plasma target‐controlled infusions for both adults and children. Current models explore covariates beyond age (maturation) and weight (size). Fat mass is now incorporated into some propofol models [30, 73, 74]. A mechanism‐based pharmacodynamic model for propofol‐induced changes in cardiovascular variables (mean arterial pressure, peripheral resistance, stroke volume, and heart rate) has been explored [75]. These innovations support assessment of drug efficacy beyond randomized controlled trials [76], and clinical drug development using a target concentration approach [18].

### 4.2 Infusion Pumps

The characterization of propofol pharmacokinetics during infusion [77] allowed early use of infusion devices [78] to deliver the drug that achieves a concentration associated with anesthesia (e.g., a target concentration of 4 μg/mL) [77]. An infusion pump, specifically designed for propofol (Diprifusor; Graseby Medical Ltd., Hertfordshire, UK) [21] was marketed, and although the pump never gained traction in the USA, it proved popular elsewhere and paved the way for further pump development. Performance evaluation of pediatric PKPD models incorporated into pumps was undertaken [79]. Plasma concentrations at which patients were arousable and decrement time estimations allowed better clinical utility. Simulation programs (e.g., Tivatrainer, https://www.tivatrainer.com) have been published, allowing practitioners to observe propofol concentration and effect over time, allowing familiarization and prediction of events.

Target‐controlled infusion pumps are now commercially available and are supplied with preloaded and activated pharmacokinetic parameter sets for drugs such as propofol as well as for remifentanil, sufentanil, and dexmedetomidine. Publication of universal models (models that encompass an age range from neonates to the elderly) for propofol [32] and remifentanil [80] is available, obviating the need for the somewhat bewildering array of pediatric parameter sets of the past. These pumps have display panels that allow clinicians to track plasma and effect site concentration, including estimation of decrement times. Safety features are designed to prevent dosing errors. Dose determination, particularly in the obese undergoing anesthesia, is becoming increasingly complex. Programmed target‐controlled infusion pumps are perhaps the most error‐free method of delivering the appropriate dose in children [81].

Pumps are a direct result of propofol introduction for maintenance anesthesia. Both plasma and effect site targeting are possible with these pumps. Effect site monitoring is a direct result from our need to measure the propofol response (pharmacodynamics).

## 5 Monitoring of Effect

Propofol is administered to achieve a specific effect (the target effect). However, titration of dose based on clinical criteria is challenging because degree of consciousness can only be assessed during sedation. Clinicians often evaluate the patient's responsiveness using verbal stimuli, movement, or vital signs to monitor anesthesia effectiveness. These variables serve as indirect indicators of anesthetic depth and poorly reflect neurophysiological effects on the brain. They do not discriminate between the hypnotic and analgesic components of general anesthesia [82, 83].

The application of electroencephalography (EEG) as a measure of anesthetic depth was proposed by Gibbs et al. in 1937 [8]. However, its integration into routine clinical practice occurred in the early 1990s with the advent of processed EEG monitors such as the Bispectral Index (BIS), spectral entropy, and auditory evoked potential monitors [84]. These devices simplified EEG data analysis to generate a numerical value correlating with consciousness levels. The EEG was used for thiopental pharmacokinetic exploration initially [85, 86], but the clinical need for propofol infusion pharmacodynamic understanding overtook that of barbiturate coma.

Cerebral monitoring has facilitated the recognition of PD between‐patient variability and has enabled the individualization of dosing regimens based on real‐time EEG changes [87]. Moreover, this real‐time measurement of EEG changes has led to the development of pharmacokinetic–pharmacodynamic (PKPD) models of propofol, which, when integrated into intelligent infusion pumps (target‐controlled infusion), facilitated dose adjustments to target concentrations at the effect site, thereby improving the control of the desired effect [88]. Furthermore, having EEG indices for hypnotic effect has propelled advancements in “closed‐loop” delivery systems, allowing for the automated administration of propofol to achieve a desired electroencephalographic target, such as a BIS of 50, rather than focusing on a particular target plasma concentration or effect site concentration [89].

Propofol acts as an agonist of the gamma‐aminobutyric acid subtype A (GABA_A_) receptor, which is distributed across the cerebral cortex, thalamus, brainstem, striatum, and spinal cord [90]. EEG primarily records electrical phenomena occurring in the cerebral cortex. Commercially available anesthesia monitors capture these signals from the anterior (frontoparietal) region of the brain using adhesive skin sensors. Typically, the EEG of an awake subject is characterized by a predominance of high‐frequency, low‐amplitude waves. Propofol induces dose‐dependent changes in the EEG, affecting the frequency, amplitude, and localization (anteriorization of alfa waves) of electroencephalographic waves [90].

Different anesthetic agents can produce distinct electroencephalographic patterns corresponding to their mechanisms of action in the brain. The typical frontal electroencephalographic pattern observed during propofol anesthesia is characterized by slow delta oscillations (0.1–4 Hz) and alpha oscillations (8–12 Hz) [90, 91]. The slow delta oscillations represent hyperpolarization of the thalamus and cortex, accompanied by reduced excitatory inputs from the brainstem to the thalamus and cortex. Propofol overdose may lead to burst suppression in the EEG, manifesting as alternating bursts of high activity associated with electrical activity suppression (flat EEG) [90, 91]. Conversely, signs of awakening are indicated by the disappearance of frontal alpha waves and the emergence of higher frequency beta waves (13–25 Hz) [90, 91].

Exploration of EEG patterns in children has paralleled propofol use in children, particularly in neonates and infants. EEG patterns change dramatically with age and neurological development [87]. While slow delta oscillations are present in children of all ages, alpha oscillations emerge around 4 months of age. The power of alpha oscillations increases during infancy, peaking around 10 months [92]. Most commercially available indices do not incorporate these developmental changes into their algorithms, limiting their utility, especially in patients younger than 1 year of age [93]. The challenges around EEG in infants may lie in the subtle difference between awake and asleep states in the cortical area, rather than in the lack of an age‐specific algorithm.

EEG‐guided anesthesia in children has demonstrated utility in reducing propofol consumption and preventing excessive sedation, leading to quicker recovery [87]. Characterization of anesthetically induced electroencephalographic oscillations in children underscores the importance of developing age‐dependent strategies for appropriately monitoring the brain states during general anesthesia [92].

Cerebral monitors enabled practitioners to target an effect rather than a drug concentration. Dose, determined by pharmacokinetics, can be adjusted to achieve an effect site concentration that achieves that the targeted effect. This practice is the basis for individualizing dose in much of current clinical pharmacology (Table 2). Use of propofol in target‐controlled infusions is a practical demonstration of this practice.

**TABLE 2:** The target concentration intervention approach involves steps to predict the dose required to achieve and maintain a target effect in an individual; clearance (CL), volume of distribution (*V*), maximum effect (*E*_MAX_), and concentration at 50% of maximum response (*C*_50_) and are the key drug parameters required to make a pharmacologically based dose prediction. These are incorporated into target‐controlled infusion (TCI) pumps. A rational approach for clinical medicine is to establish the target effect using the population values of these four key parameters. Individual differences can be predicted using covariate data (e.g., age, weight that are entered into TCI pumps) and observed patient responses.

1. Choose the target effect (TE) e.g., a bispectral index score 50 units (scale 0–100)	
2. Use *E*_MAX_ and *C*_50_ to predict the target concentration (TC)	
3. Use *V* to predict the loading dose (LD) and clearance (CL) to predict the maintenance dose rate or infusion rate (MDR) LD = target concentration × *V* MDR = target concentration × CL	
4. Measure response (e.g., BIS change)	
5. Revise target concentration. This is a key component for closed‐loop systems, but is achieved manually in current clinical practice using TCI pumps	
6. Measure drug serum concentrations	These steps comprise an essential part of the target concentration intervention strategy for individualization in clinical pharmacology but are not part of TCI pumps
7. Revise *V* and CL	
8. Return to Step 3	

## 6 The Target Concentration Approach

**FIGURE 4:**
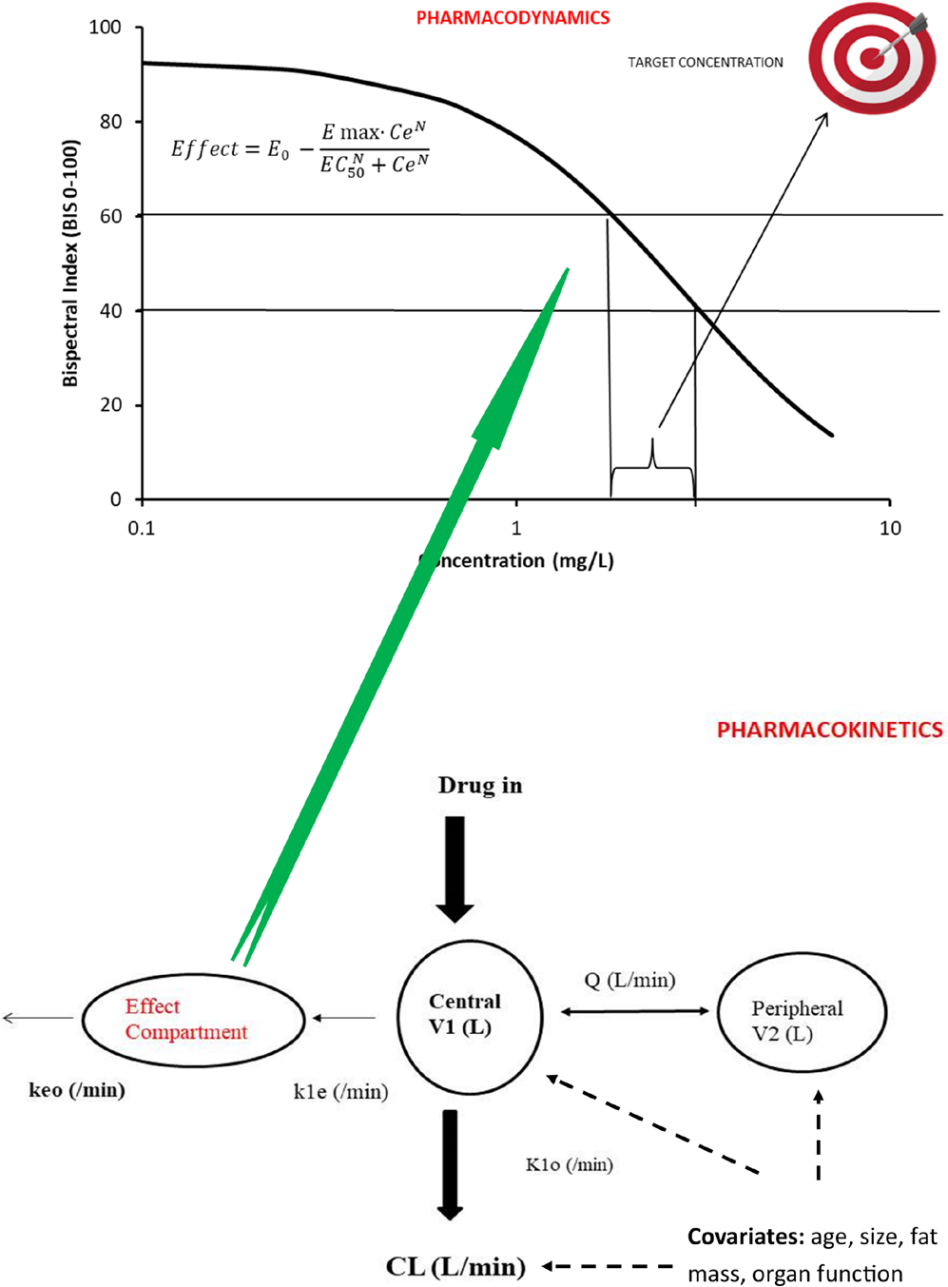
Principles behind the target concentration strategy are shown diagrammatically. The upper panel shows a concentration–response for propofol and BIS at the effect site, determined using a sigmoidal *E*_MAX_ model with those key parameters (*C*_50_, *E*_MAX_). The BIS target range of 40–60 is associated with a target concentration of 1.8–3.5 mg/L. Pharmacokinetic knowledge (lower panel) is then used to determine a dose that achieves this target concentration in the effect compartment. A two‐compartment pharmacokinetic model is shown in this example.

The target effect is the goal of drug treatment. This target effect is associated with a target concentration. The pharmacokinetic model can be used to calculate a dose [94] that achieves the target concentration, a process known as the target concentration strategy. The target concentration is the concentration desired in the plasma (*C*_p_) or at the effect site (*C*_e_). The plasma and effect site concentrations are the same at steady state. The effect site concentration–response relationship of the drug will differ with age, pathology, drug interactions, and stimulus (Figure 4). The target concentration will differ depending on the magnitude of the desired effect.

The concentration–response relationship at the effect site is often described using the *E*_MAX_ equation; this versatile equation was used to describe the oxygen dissociation curve [95], a physiology well understood by anesthesia practitioners since the introduction of pulse oximetry worldwide in the late 1980s. There is a delay between plasma concentration (*C*_p_) and concentration in the effect site (*C*_e_) described using a single first‐order parameter (*T*_1/2_keo). This effect compartment concept was first introduced into anesthesia for neuromuscular blocking drugs [9, 96], and rapidly became an important component of parameter sets used in TCI pumps. It is, after all, the concentration in the effect site that is the target.

This *T*_1/2_keo parameter was missing from early pediatric pharmacokinetic analyses of propofol [17]. Estimation requires both PK and PD data to be collected simultaneously and parameters for both PK and PD models estimated together. An alternative method (*T*_peak_ methodology) involving measurement of the time to maximum effect was used [97]. An allometrically standardized *T*_1/2_keo (i.e., *T*_1/2_keo_ADULT_) of 2.38 (95% CI 1.84, 3.16) for propofol is described [36]. Exploration of this link parameter was important for safe delivery of propofol during anesthesia. If incorrect, then the administered dose would be wrong. PKPD modeling of drugs now inevitably involves exploration of delayed effects; a concept originally explored for the neuromuscular blocking drugs with early extension to propofol because of clinical need.

Nowhere is this target concentration strategy better exemplified than that with the clinical use of propofol by anesthetists. Algorithms built on PKPD models permit target‐controlled infusions for both adults and children. A propofol concentration in the effect compartment (*C*_e_) of 2–3 μg/mL is an appropriate target for sedation and 4–6 μg/mL is adequate for anesthesia. Anesthesiologists use the target concentration strategy every day in clinical practice.

## 7 The Supraglottic Mask Airway

The laryngeal mask airway [11] was a wondrous device for anesthesiologists practicing in the 1980s. Avoidance of endotracheal intubation doomed practitioners to either hold a face mask or devise methods to secure that face mask in a manner that maintained a clear airway (e.g., Clausen head harness, chin strap). The face mask ensured practitioners developed a firm handshake but tied those same practitioners to the patient, unable to perform other functions. Use of the laryngeal mask airway and use of propofol rose in parallel. Reports that propofol had greater depression of pharyngeal and laryngeal reactivity than seen with thiopental [13] supported the synchronous rise in popularity of both device and drug.

## 8 Other Influences on Anesthesia Practice

The introduction of propofol changed the way we practice pediatric anesthesia and continues to do so. The lipid content of the emulsion carrier and propofol's depression of mitochondrial function may play a role in propofol infusion syndrome [98] and has influenced rates for long duration infusion. Concerns about neurotoxicity to the infant brain have generated decades of investigation [99].

Propofol had an unexpected direct antiemetic effect [100], an important benefit for patients anesthetized with propofol infusion. While propofol remains widely used for postoperative emergence delirium, investigations are ongoing to determine if infusion can reduce postoperative cognitive dysfunction more than that observed with inhalation anesthesia [101].

The nutrient rich emulsion carrier may also encourage bacterial contamination, and such worries have driven aseptic practices at the workspace and discard of vials or ampoules after a limited period following opening [102]. Pain associated with intravenous injection into small veins has altered how we administer the drug and use of supplementary drugs (remifentanil, lidocaine) to lessen that pain. It has also encouraged research into alternative carrier substances (e.g., cis‐propofol).

The environmental impact of anesthetic gases and vapors is substantial. The health sector contributes approximately 4.4% of carbon emissions throughout the world [103]. Induction using inhalation agents and nitrous oxide remains popular for children but there is a move away from nitrous oxide to lesson environmental impact [103]. The inhalational vapors remain troublesome with their global warming potential. Efforts to achieve a more sustainable practice such as volatile capture have been proposed [104]. TIVA, based on propofol, may be more environmentally friendly [105, 106], but this surmise is unsubstantiated. It is unknown if waste intravenous drugs (including those used in anesthesia) are better for the environment than halogenated agents. TIVA may seem eco‐friendly, but a comprehensive analysis of those drugs is required, and that analysis should assess the environmental impact of manufacturing, transportation, installation, and usage [107].

## 9 Conclusions

The introduction of propofol has shaped pediatric anesthesia practice. Propofol introduction improved our understanding of pharmacology, initiated new research approaches for intravenous drug study, and led to the use of infusion pumps for the maintenance phase of anesthesia. Propofol pharmacodynamic properties encouraged supraglottic airway use and became the primary treatment for postoperative delirium after inhalational anesthesia. While some progress during the rise of propofol can be attributed to either plan or parallel development, others are simply random events; environmental drug impact concerns were serendipitous. The story of propofol development provided a blueprint for other intravenous drug development [3]. Use of propofol in TCI pumps is a practical example of the target concentration approach to drug therapy and clinical drug development.

## Ethics Statement

Human ethics committee approval not required.

## Conflicts of Interest

Brian J. Anderson and L. Ignacio Cortinez serve on the Editorial Board for the journal Pediatric Anesthesia.

## Data Availability

Data sharing is not applicable to this article as no new data were created or analyzed in this study.
